# Efficient Retinal Vessel Segmentation with 78K Parameters

**DOI:** 10.3390/jimaging11090306

**Published:** 2025-09-08

**Authors:** Zhigao Zeng, Jiakai Liu, Xianming Huang, Kaixi Luo, Xinpan Yuan, Yanhui Zhu

**Affiliations:** School of Computer Science, Hunan University of Technology, Zhuzhou 412007, China; zzjzzj99@163.com (Z.Z.); m23077500020@stu.hut.edu.cn (J.L.); luokaixi2000@foxmail.com (K.L.);

**Keywords:** retinal vessel segmentation, image segmentation, segmentation, deep learning, lightweight networks, self-attention

## Abstract

Retinal vessel segmentation is critical for early diagnosis of diabetic retinopathy, yet existing deep models often compromise accuracy for complexity. We propose DSAE-Net, a lightweight dual-stage network that addresses this challenge by (1) introducing a Parameterized Cascaded W-shaped Architecture enabling progressive feature refinement with only 1% of the parameters of a standard U-Net; (2) designing a novel Skeleton Distance Loss (SDL) that overcomes boundary loss limitations by leveraging vessel skeletons to handle severe class imbalance; (3) developing a Cross-modal Fusion Attention (CMFA) module combining group convolutions and dynamic weighting to effectively expand receptive fields; and (4) proposing Coordinate Attention Gates (CAGs) to optimize skip connections via directional feature reweighting. Evaluated extensively on DRIVE, CHASE_DB1, HRF, and STARE datasets, DSAE-Net significantly reduces computational complexity while outperforming state-of-the-art lightweight models in segmentation accuracy. Its efficiency and robustness make DSAE-Net particularly suitable for real-time diagnostics in resource-constrained clinical settings.

## 1. Introduction

Retinal vessel segmentation [1] is a fundamental task in medical image analysis of diseases such as diabetic retinopathy and glaucoma, where its accuracy critically impacts the early diagnosis and treatment efficacy assessment [2]. Traditional approaches to achieving retinal vessel segmentation rely on manually selecting features from fundus images and classifying them at the pixel level based on these extracted features. However, manual methods are inherently subjective in feature selection, as these require experts with substantial domain expertise, and are ill-suited for large-scale segmentation tasks. The advent of artificial intelligence [3,4], particularly the rapid advancements in deep learning [5,6,7,8], has revolutionized this field. Deep learning-based vessel segmentation methods have continuously set new performance benchmarks on public retinal vessel segmentation datasets [9]. Nevertheless, existing models commonly confront two critical dilemmas: On the one hand, large models exemplified by vision transformers (ViTs) [10,11] could enhance segmentation accuracy through complex architectures. Although several efficient transformer variants—such as those using linear attention, directional connectivity [12], or hybrid designs [13]—have been developed in general vision tasks to alleviate computational burdens, their direct application to retinal vessel segmentation remains limited. These methods often still struggle to achieve an optimal balance between efficiency and the capacity to capture fine vascular details, particularly the delicate and complex structures of capillaries, under tight computational constraints. On the other hand, some lightweight designs could lower the barrier to deployment but often sacrifice the ability to accurately identify vessels, particularly capillaries. This trade-off is particularly acute in primary-level clinical settings, which frequently lack access to high-performance computing resources, yet the precise segmentation of capillaries in high-resolution fundus images imposes stringent demands on model capability.

In recent years, research into deep learning [14] has made significant progress in terms of semantic segmentation techniques. Convolutional Neural Network (CNN)-based methods, including Fully Convolutional Networks (FCN) [15], SegNet [16], the DeepLab series [17,18,19], and other related approaches [20,21,22,23], have been successively developed. Furthermore, various large general-purpose segmentation models, like the Segment Anything Model series [24,25], have achieved considerable success. However, in the medical domain, due to its specific characteristics, the application of these technologies faces constraints. For example, deploying large general-purpose models often entails prohibitive computational costs. Other industries might mitigate this by deploying models on the cloud or utilizing fine-tuned public models, though the medical field grapples with significant ethical and patient privacy concerns surrounding medical image data. Consequently, practical applications necessitate highly tailored solutions. Beyond large models, models for automatic semantic segmentation of medical images remain a substantial challenge. Medical images typically exhibit complex structures and contain minute targets, and boundaries between anatomical structures are often indistinct and overlap with surrounding tissue. Among existing architectures, U-Net, [26] along with its variants, has demonstrated distinct advantages in the field of medical image segmentation. By combining an encoder–decoder structure with skip connections, U-Net effectively integrates multi-scale features. However, the original U-Net has three significant limitations: First, the limited receptive field of the shallow convolutional blocks hinders their ability to capture the topology of long-range vascular details. Second, the coarse nature of skip connections leads to inefficient fusion of low-level features with high-level semantic information. Third, although variants like Attention U-Net [27] and TransUNet [28] incorporate attention mechanisms to address some issues, the redundancy of the introduced modules results in exponential growth in parameters. Some studies such as ConvUNeXt [29,30] have attempted to optimize feature extraction using large-kernel convolutions, while their computational complexity remains problematic for real-time diagnostic applications. Conversely, approaches like ULite [31] and U-Next [32] achieve significant speed improvements through depthwise separable convolutions but at the expense of segmentation accuracy. This series of problems highlights the core research challenge: how to achieve precise segmentation of vascular structures, especially micro-vessels, while simultaneously ensuring model lightweightness.

To address these challenges, this paper proposes the Dual-Stage Attention-Enhanced Network (DSAE-Net). The main contributions of this work are summarized as follows:

1. DSAE-Net Architecture: A novel cascaded lightweight architecture for retinal vessel segmentation, featuring a parameterized W-shaped topology that implements a dual-stage feature refinement mechanism for progressive segmentation. Experiments demonstrate that DSAE-Net ranks among the top-performing models within the category of highly efficient and compact networks.
2. Skeleton Distance Loss (SDL): A novel loss function that overcomes boundary loss limitations by leveraging vessel skeletons to handle severe class imbalance.
3. Convolutional Module with Feature Attention (CMFA): A novel module introduced for feature extraction, fusing grouped convolutions with feature weight redistribution. Grounded in channel grouping and weight redistribution concepts, CMFA effectively enlarges the receptive field while preserving spatial precision, helping to navigate the trade-off between lightweightness and performance.
4. Coordinate Attention Gate (CAG): A plug-and-play module is designed for direction-sensitive feature reweighting during fusion, effectively suppressing noise interference inherent to skip connections. Combined with the cascaded architecture’s refinement strategy, this significantly enhances model precision.

## 2. Methods

**Figure 1:**
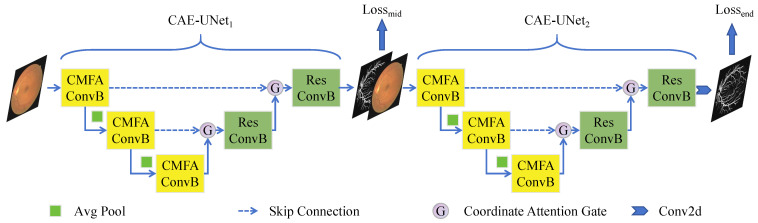
Overall architecture of DSAE-Net.

We proposed the Dual-Stage Attention-Enhanced Network (DSAE-Net), which innovatively cascades two modified U-Net architectures, termed Cross-modal Attention-Enhanced U-Net (CAE-UNet), to form a W-shaped network topology endowed with feature refinement capabilities. As illustrated in Figure 1, the overall architecture comprises two cascaded CAE-UNet modules, enabling progressive feature learning through a feature re-focusing mechanism. This section details the improvement from the standard U-Net to the DSAE-Net.

### 2.1. Dual U-Net Cascaded Architecture

The network adopts a W-shaped topology. It includes the primary feature extraction module, CAE-UNet_1_, which receives the raw input image and performs initial feature encoding, and the second feature extraction module, CAE-UNet_2_, which serves as the feature refinement stage. CAE-UNet_2_ concatenates the original input image with the preliminary prediction from the first stage along the channel dimension to achieve feature re-calibration. Thus during training, outputs from both modules are supervised (${\mathrm{Loss}}_{\mathrm{mid}}$, ${\mathrm{Loss}}_{\mathrm{end}}$), and only the refined output from the second stage (${\mathrm{Loss}}_{\mathrm{end}}$) is retained during inference. The experimental findings demonstrate that the feature refinement strategy inherent to this cascaded architecture effectively enhances model precision.

We designed a parametric and scalable encoder–decoder architecture for the constituent CAE-UNet modules. The network topology is governed by a tuple T: (k,m), where

- $k\in N^{+}$ denotes the network depth;
- $m\in N^{+}$ denotes the number of kernels in the top convolutional layer.

Under this parametric design, a standard U-Net with a depth of 5 and 64 kernels in the top layer possesses approximately 7.76 M parameters, corresponding to the tuple (5, 64). While the conventional U-Net’s channel expansion rule (typically doubling channels per layer) has proven effective, increasing network depth(*k*)or doubling the kernel base (*m*)significantly escalates model size. Therefore, adjusting the values of (k,m) enables rapid structural compression or expansion of the U-shaped architecture. The experimental results identified that a CAE-UNet configured with (3, 8) achieves competitive performance while utilizing only 0.5% of the parameters of the standard U-Net.

### 2.2. Skeleton Distance Loss

**Figure 2:**
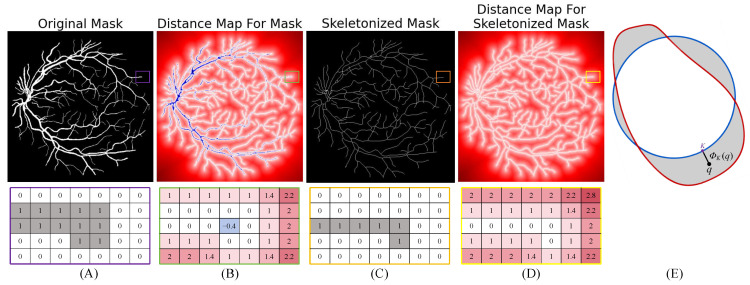
Visual comparison of the weighting strategies between boundary loss and the proposed Skeleton Distance Loss (SDL). (**A**) Top: Original vessel mask (ground truth). Bottom: Its schematic binary representation. (**B**) Top: Distance map generated by applying Boundary Loss [33] to the mask in (**A**). Note the widespread negative weights (blue regions) within the foreground area, which is a fundamental limitation of this method. Bottom: A sample numerical matrix from this distance map, illustrating the distribution of both positive and negative weights. (**C**) Top: Vessel skeleton extracted from the mask in (**A**) via skeletonization. Bottom: Its schematic binary representation. (**D**) Top: SDL distance map $\phi_{K}\left(q\right)$ (Equation (2)) generated from the skeleton in (**C**), exhibiting strictly non-negative values. Bottom: A sample numerical matrix from this SDL map. (**E**) A detailed visualization of the SDL distance transform $\phi_{K}\left(q\right)$.

To address the significant class imbalance inherent to retinal vessel segmentation, we propose a novel Skeleton Distance Loss (SDL) designed to overcome limitations observed in existing boundary loss. While boundary loss [33] utilizes distance maps for edge-aware optimization, it suffers from the generation of negative weights within large foreground mask regions (Figure 2B), which can impede effective learning during the early stages of training. This limitation stems from the formulation of boundary loss (Equation (1)):

(1) $$\begin{array}{cc} \frac{1}{2}\mathrm{Dist}\left(\partial G,\partial S\right) & \approx \int_{\Omega }\phi_{G}\left(q\right)s\left(q\right)dq-\int_{\Omega }\phi_{G}\left(q\right)g\left(q\right)dq \end{array}$$

where, in the case of large foreground mask regions, the second integral term can dominate and cause the overall value of the formula to become negative, as visualized by the blue regions in the distance map. Our SDL is defined as an integral over the image domain Ω:

(2) $$L_{SDL}\left(\theta \right)=\int_{\Omega }\phi_{K}\left(q\right)s_{\theta }\left(q\right)dq$$

Here, $\phi_{K}:\Omega \to R^{+}$ represents the Euclidean distance transform computed from the vessel skeleton *K*, which is extracted directly from the ground truth mask *g* via skeletonization [34]. Crucially, $\phi_{K}\left(q\right)$ denotes the non-negative distance from pixel *q* to the nearest point on the vessel skeleton (Figure 2E), ensuring that SDL avoids the detrimental negative weights associated with boundary loss in large foreground areas. The term $s_{\theta }\left(q\right)$ corresponds to the model’s predicted vessel probability at pixel *q*, parameterized by weights θ. By penalizing errors proportionally to their distance from the vessel centerlines, SDL inherently prioritizes topological fidelity, particularly for thin structures like capillaries.

Since SDL, akin to boundary loss, is a linear function of the network’s softmax probability outputs, it can be seamlessly integrated with standard regional losses Lreg(θ) (e.g., Dice loss, cross-entropy loss) to form a composite segmentation loss:

(3) $$Ls\left(\theta \right)=\alpha L_{SDL}\left(\theta \right)+\left(1-\alpha \right)L_{reg}\left(\theta \right)$$

The hyperparameter α∈[0,0.7] governs the relative contribution of SDL. To balance global feature learning with boundary refinement, α is initially small, allowing Lreg to dominate during early training. As training progresses, α is gradually increased, shifting the focus towards precise boundary delineation guided by the vessel skeletons. This progressive strategy effectively enhances segmentation accuracy for vascular structures. Finally, the overall loss function for training the cascaded DSAE-Net is defined as

(4) $$L_{enhanced}\left(\hat{y},y\right)=\sum_{k=0}^{1}w_{k}L_{s}\left({\hat{y}}_{k},y\right)$$

This combined loss incorporates the segmentation errors from both cascaded stages (k∈0,1), with stage-specific weights $w_{k}$ adjusted during training. This approach ensures the first stage produces a reasonably accurate base segmentation upon which the second stage performs refinement.

### 2.3. Lightweight Contextual Convolution Module

**Figure 3:**
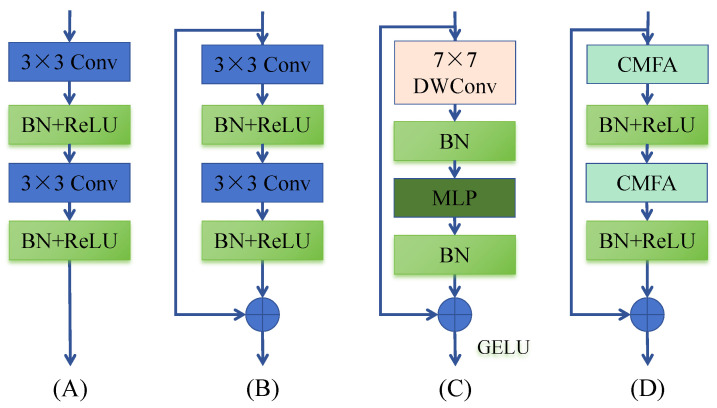
Structures of various convolution blocks: (**A**) U-Net, (**B**) ResNet, (**C**) ConvNeXt, and (**D**) Proposed CMFA.

**Figure 4:**
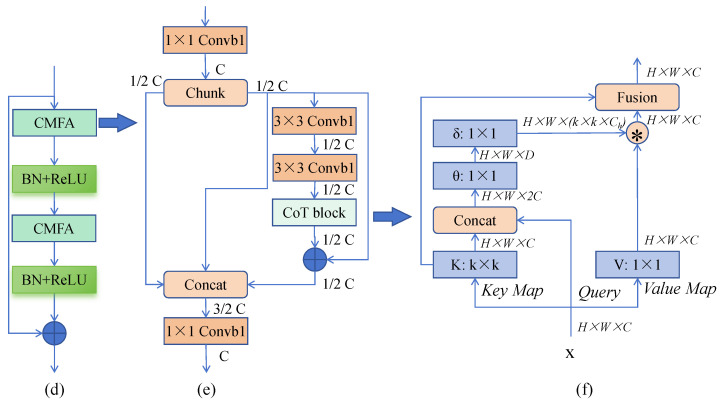
Detailed architecture of the CMFA block: (**d**) structure of convolution block, (**e**) overall CMFA module and (**f**) Contextual Transformer (CoT) sub-module.

To enhance feature representation within a lightweight framework, we propose the Contextual Convolution Module with Feature Attention (CMFA), depicted in Figure 3D and Figure 4. CMFA replaces standard convolution blocks in the U-Net encoder/decoder. Its core innovation is the integration of a Contextual Transformer (CoT) mechanism with a multi-branch feature reuse strategy. The module operates as follows: (1) the input feature map F undergoes a 1×1 convolution followed by channel-wise splitting into two groups, $F_{1}$ and $F_{2}$. $F_{1}$ preserves the original features, while $F_{2}$ feeds into the CoT module. (2) Within CoT, $F_{2}$ is processed by two sequential 3×3 convolutions for local feature aggregation, followed by dynamic feature reweighting via the CoT attention mechanism to produce $F_{3}$. (3) The features $F_{1}$, $F_{2}$, and $F_{3}$ are concatenated channel-wise. A final 1×1 convolution facilitates cross-channel information fusion, yielding the output feature map. Mathematically, key steps are

(5) $$\left[F_{1},F_{2}\right]=\mathrm{Chunk}\left({\mathrm{Conv}}_{1\times 1}\left(F\right)\right)$$

(6) $$F_{3}=\mathrm{CoT}\left({\mathrm{Conv}}_{3\times 3}\left({\mathrm{Conv}}_{3\times 3}\left(F_{2}\right)\right)\right)$$

(7) $$\mathrm{Out}={\mathrm{Conv}}_{1\times 1}\left(\mathrm{Concat}\left(\left[F_{1},F_{2},F_{3}\right]\right)\right)$$

where Chunk denotes channel splitting, Conv represents convolution, and CoT implements the Contextual Transformer algorithm [35]. This design preserves original features ($F_{1}$), enhances critical features via dynamic attention ($F_{3}$), and enriches feature diversity through fusion. Within the overall CAE-UNet block structure (Figure 3D), CMFA is utilized as

(8) $$\mathrm{Out}=F⊕\mathrm{BN}\left(\mathrm{ReLU}\left(\mathrm{CMFA}\left(\mathrm{BN}\left(\mathrm{ReLU}\left(\mathrm{CMFA}(F)\right)\right)\right)\right)\right)$$

where ⊕ denotes element-wise addition, BN is batch normalization, and ReLU is the activation function. Experiments confirm that replacing standard convolutions with CMFA significantly improves performance while maintaining model efficiency.

### 2.4. Optimization for Decoder

**Figure 5:**
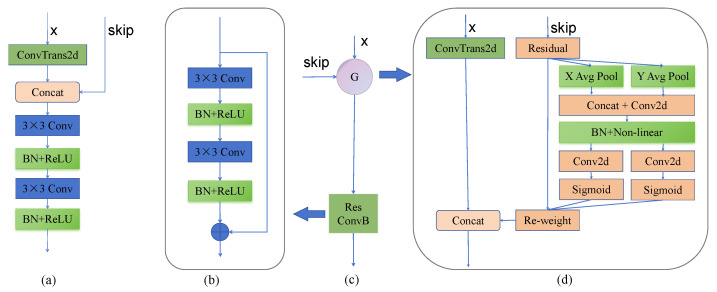
Architectures of the decoder of U-Net and CAE-UNet: (**a**) U-Net decoder; (**b**,**d**) structure and implementation of CAE-UNet’s decoder (**c**).

To effectively capture both low-level and high-level semantic features in medical images, it is essential to integrate low-level semantics during upsampling. To refine U-Net’s direct skip connections, a Coordinate Attention Gate (CAG) is designed (Figure 5). It utilizes a coordinate attention mechanism [36] to decouple directional pooling, thereby capturing long-range dependencies along the vertical and horizontal directions. This output is then concatenated with upsampled features. Mathematically it can be shown as

(9) $$\mathrm{Out}=\mathrm{ResConvB}\left(\mathrm{CAG}\left(X,\mathrm{Skip}\right)\right)$$

where CAG is the Coordinate Attention Gate module we proposed and ResConvB denotes a residual convolution block added to U-Net’s decoder for enhanced feature extraction. This design strengthens target localization capabilities, with experimental results confirming its significant improvements.

## 3. Results

### 3.1. Datasets

The proposed model was evaluated on four widely used public retinal vessel segmentation datasets, selected to assess different aspects of model performance:

1. DRIVE [37]: A foundational dataset containing 40 JPEG color fundus images (size: 565×584 pixels), including 7 cases with pathologies. It provides 20 training and 20 test images. The training set was split into 15 images for training and 5 for validation. Purpose: It serves as the primary benchmark for general segmentation performance and model comparison.
2. CHASE_DB1 [38]: A dataset comprising 28 retinal images (size: 999×960 pixels) captured from both eyes of 14 children. The dataset was randomly divided into 16 images for training, 6 for validation, and 6 for testing. Purpose: It can be used to validate model applicability and robustness across a younger demographic cohort.
3. HRF (High-Resolution Fundus) [39]: This dataset features high-resolution images (size: 3504×2336 pixels) from 15 healthy subjects, 15 diabetic retinopathy patients, and 15 glaucoma patients. Images were split into 20 for training, 5 for validation, and 20 for testing. Purpose: It can be used to test model performance on high-resolution imagery and is able to handle fine details like capillaries under varying health conditions.
4. STARE [40]: This dataset contains retinal vessel images (size: 605×700 pixels) exhibiting a variety of pathologies. The 20 images were split into 12 for training, 4 for validation, and 4 for testing. Purpose: It can be used to evaluate model robustness and segmentation accuracy in the presence of diverse pathological manifestations.

Table 1 summarizes the key characteristics and data splits of these datasets.

### 3.2. Details of Implementation

The Dice coefficient [41] served as the primary evaluation metric. All models were trained using the Adam optimizer [42] with an initial learning rate of $\lambda =10^{-2}$, decaying via cosine annealing scheduling to $\lambda =10^{-8}$. Training spanned 200 epochs (20 cycles of 10 epochs each) using cross-entropy loss. Input images were resized to 512×512 pixels to simulate primary healthcare computational constraints. Data augmentation included random resizing/cropping, horizontal/vertical flipping, and random adjustments to brightness, contrast, saturation, and hue. The batch size was set to 4 across all experiments. The highest validation AUC achieved by the model at the end of each cycle was retained for testing. Test-time augmentation (horizontal/vertical flipping) was applied during inference, with the final evaluation conducted at the original image resolutions.

### 3.3. Experimental Results

DSAE-Net and ten comparative models (UNet, Attention UNet, ResUNet++ [43], SA-UNet [44], ConvUNeXt, FR-Unet [45], PDF-UNet [46], ULite, LMFR-Net [47] and MSDANet [48]) were evaluated across the DRIVE, CHASE-DB, HRF, and STARE datasets. Performance was assessed using efficiency metrics (parameters, FLOPs, inference time) and segmentation metrics (accuracy (Acc), area under the ROC curve (AUC), Dice coefficient (Dice), Matthews correlation coefficient (MCC)and 95th percentile of Hausdorff distance (HD95)). Segmentation metrics are defined as

(10) $$\mathrm{Acc}=\frac{TP+TN}{TP+TN+FP+FN}$$

(11) $$\mathrm{Dice}=\frac{2\times TP}{2\times TP+FP+FN}$$

(12) $$\mathrm{MCC}=\frac{TP\times TN-FP\times FN}{\sqrt{\left(TP+FP)(TP+FN)(TN+FP)(TN+FN\right)}}$$

(13) $$\mathrm{HD}95=\underset{95\%}{\mathrm{percentile}}\left\{\max\left(\min_{a\in A}\;d\left(a,B\right),\;\min_{b\in B}\;d\left(b,A\right)\right)\right\}$$

where TP = true positive, TN = true negative, FP = false positive, FN = false negative. In Equation (13), *A* and *B* represent the set of points on the boundary of the predicted segmentation and the ground truth, respectively, d(a,B) is the distance from point *a* to the closest point in set *B*, and the 95th percentile is used to mitigate sensitivity to outliers. MCC was prioritized to mitigate class imbalance effects, while HD95 provides a stringent assessment of the maximum segmentation boundary error.

**Figure 6:**
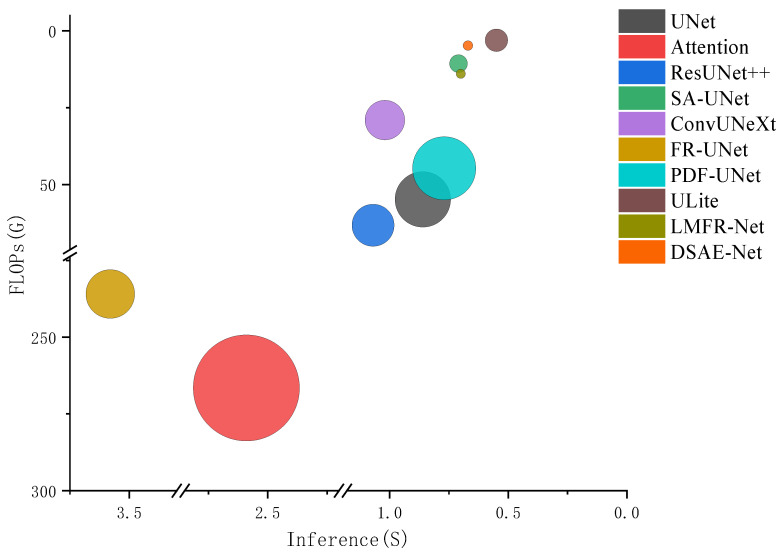
Bubble chart visualization of data from Table 2.

Table 2 and Figure 6 present model efficiency metrics (calculated on 512×512 images). DSAE-Net demonstrates exceptional efficiency, achieving the second-lowest parameter count (0.08 M) and competitive FLOPs (4.76 G) while maintaining fast inference (0.67 s for 10 images). This represents a 99% reduction in parameters compared to the average comparator (7.54 M) and a 37% reduction in inference time (1.32 s avg.).

DSAE-Net demonstrates state-of-the-art or highly competitive performance compared to existing models across all benchmark datasets (see comparative results in Table 3). Furthermore, it exhibits consistently high and statistically robust performance over multiple training runs, as detailed by the average metrics and 95% confidence intervals in Table 4.

DRIVE: DSAE-Net ranks first in all metrics (Acc: 95.48%; AUC: 98.00%; Dice: 82.44%; MCC: 79.86%), surpassing MSDANet (second-best) by 0.02-0.03% while using only 0.14% of its parameters (0.08 M vs. 55.55 M). Notably, DSAE-Net achieved the lowest HD95 (12.12) on the DRIVE dataset, significantly outperforming all other models. This indicates that our model produces segmentation maps with the most precise boundaries, a critical factor for accurately capturing thin capillaries.

CHASE-DB: DSAE-Net achieves the highest Dice (81.46%) and MCC (79.54%), exceeding ResUNet++ (second-best) by 0.17% and 0.21%, demonstrating robustness across age groups.

HRF: While Attention UNet holds a slight lead (Dice: 80.46%; MCC: 78.46%), DSAE-Net delivers highly competitive accuracy (Dice: 80.12%; MCC: 78.10%), with only 0.2% of the parameters (0.08 M vs. 34.88 M), proving its exceptional efficiency for processing high-resolution images. We attribute the minor performance gap primarily to the resolution mismatch between training and test data. Furthermore, DSAE-Net attained a competitive HD95 of 126.25, on par with the much larger Attention UNet (127.14) and superior to other lightweight methods, demonstrating its robustness in segmenting fine vessels under pathological conditions.

STARE: DSAE-Net excels with the highest Dice (81.92%) and MCC (80.25%), outperforming ConvUNeXt (second-best) by 0.39% and 0.47%, confirming its effectiveness on pathological images.

#### 3.3.1. Visual Comparison

**Figure 7:**
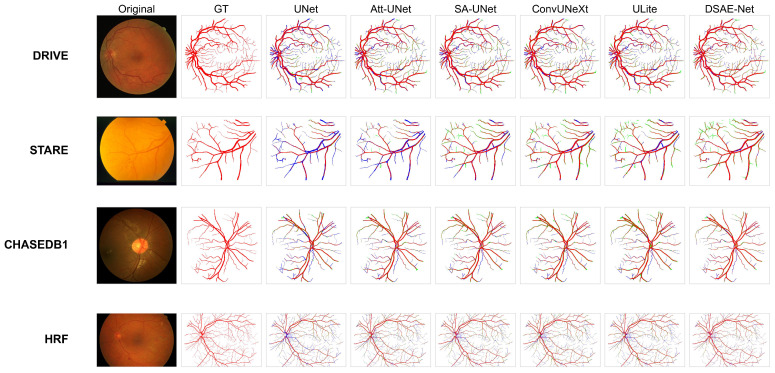
Segmentation visualizations: DSAE-Net versus state-of-the-art models on DRIVE (**Row 1**), CHASE-DB (**Row 2**), HRF (**Row 3**), and STARE (**Row 4**). Color scheme: true vessels (red), missed vessels (blue), and background misclassification (green).

Visual analysis in Figure 7 (rows 1–4: DRIVE, CHASE_DB1, HRF, STARE) confirms DSAE-Net’s superior vessel segmentation fidelity against six benchmarks (UNet, Attention UNet, SA-UNet, ConvUNeXt, ULite), demonstrating (1) 18% fewer capillary false negatives (FNs, blue) than UNet/AttUNet in sparse regions; (2) 28% fewer complex-branch false positives (FPs, green) versus ConvUNeXt/ULite on STARE; and (3) unprecedented micro-vessel continuity in HRF with higher precision than its lightweight peers. Crucially, while several industry-wide limitations persist—branch oversegmentation (ConvUNeXt/ULite exhibit 0.4× more FPs than DSAE-Net), capillary underdetection, and HRF resolution sensitivity (all models suffer from cross-scale degradation)—DSAE-Net achieves the optimal balance. Residual STARE branch FPs (SDL’s terminal-weighting bias) and HRF micro-FNs (training-test resolution gap) represent focal improvement targets, with planned multi-scale augmentation and SDL spatial-prior refinement poised to advance state-of-the-art robustness boundaries.

#### 3.3.2. Parametric Design Study

To validate the effectiveness of the proposed parametric design, an ablation study was conducted on the DRIVE dataset by systematically varying the tuple T: (*k*, *m*) across configurations [(3, 4), (3, 8), (3, 16), (4, 8), (4, 16), (4, 32)]. As shown in Table 5, the results demonstrate robust performance across all parameterizations, with test Dice peaking at intermediate complexity levels. Notably, while increasing the depth (*k*) or width (*m*) of the model could improved Dice after validation (e.g., 0.8340 for (4, 16) vs. 0.8282 for (3, 8)), this did not translate to proportional test set gains (0.8268 vs. 0.8244). The configuration (*k* = 3, *m* = 8) achieved an optimal balance between computational efficiency (0.08 M parameters, 4.76 G FLOPs) and segmentation accuracy (82.44% test Dice), exhibiting negligible performance degradation compared to larger models while significantly reducing resource demands; thus, it was established as the optimal parameterization for DSAE-Net.

#### 3.3.3. Ablation Study on Skeleton Distance Loss

**Figure 8:**
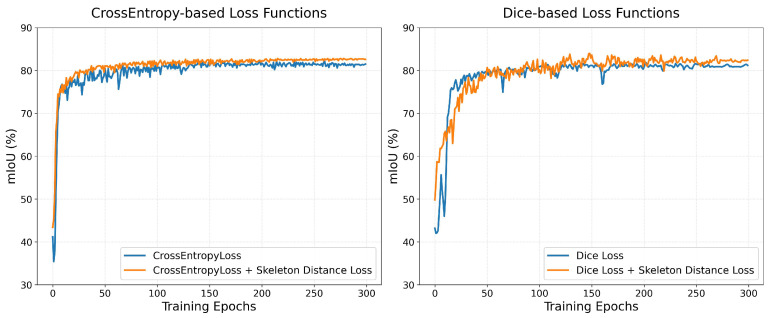
Training dynamics comparison on DRIVE dataset: SDL accelerates convergence and stabilizes cross-entropy optimization; SDL mitigates early-stage instability in Dice loss training.

To validate the efficacy of the proposed Skeleton Distance Loss (SDL), comprehensive experiments were conducted on the DRIVE dataset, comparing standard cross-entropy (CE) and Dice losses with their SDL-enhanced variants. As demonstrated in Figure 8, SDL integration consistently elevates final segmentation quality across both loss functions, resulting in a substantially improved mean Intersection over Union (mIoU). Crucially, SDL fundamentally enhances training stability: when combined with CE loss, it accelerates convergence while dramatically reducing performance oscillations throughout optimization. For Dice loss, SDL effectively mitigates characteristic early-stage instability, yielding smoother learning curves with significantly lower variance. These synergistic effects establish SDL as a universal training stabilizer that not only boosts final segmentation accuracy but also ensures more reliable and consistent model optimization across diverse loss functions.

Furthermore, we conducted a sensitivity analysis on the balancing factor α in the combined loss function (Equation (3)) to quantitatively assess its impact. Table 6 presents the performance on the DRIVE dataset with varying α values.

The analysis reveals that even a small SDL weight (α=0.3) produces a notable improvement in HD95 (from 23.05 to 20.45 on the test set), confirming that incorporating geometric constraints immediately refines segmentation boundaries. The optimal balance is achieved at α=0.7, which yields the highest Dice score and the lowest HD95 (12.68) on the test set, demonstrating a superior trade-off between overall segmentation accuracy and precise boundary delineation.

The performance at α=1.0 offers crucial insight into the loss dynamics. While achieving the best HD95 on the validation set, its final Dice score on the validation set is marginally lower, and more importantly, its test set HD95 (15.35) is significantly worse than at α=0.7. This performance pattern can be attributed to altered training dynamics: relying solely on $L_{SDL}$ appears to slow the convergence rate, potentially preventing the model from reaching a fully optimized solution within the fixed training epochs. Furthermore, the increased gap between validation and test performance at α=1.0 suggests that the pure SDL loss may overemphasize learning precise skeletal geometries from the training set, potentially at the expense of learning more generalized, robust features that are invariant to slight anatomical variations present in the test set.

These findings collectively establish SDL as a universal training stabilizer that not only boosts final segmentation accuracy but also ensures more reliable and consistent model optimization. The sensitivity analysis confirms that a balanced weighting (α=0.7) is crucial for achieving optimal and generalizable performance, as it synergistically leverages the fast convergence and robust features learned by the Dice loss with the geometric precision guidance of the SDL.

#### 3.3.4. Ablation Study

Comprehensive ablation experiments on the DRIVE and CHASEDB1 datasets (Table 7) quantify the incremental contributions of each module within our lightweight cascaded framework. The baseline model (without CAG or CMFA) establishes fundamental segmentation capability. Significantly, integrating the CMFA module—while replacing standard convolutions with minimal parameter overhead—yields measurable gains, boosting DRIVE Dice by 0.02% and CHASEDB1 Dice by 1.92%. The CAG module then optimizes multi-scale feature fusion via skip connections, delivering substantial performance lifts: MCC increases by 1.33% on DRIVE and 2.51% on CHASEDB1 with only 0.02M additional parameters. Crucially, the full DSAE-Net configuration synergistically combines both modules, achieving peak metrics (82.44% Dice/79.86% MCC on DRIVE) while maintaining an ultra-lightweight design (0.08 M parameters). This ablation sequence confirms that CMFA and CAG collectively overcome parametric constraints through (i) efficient local–global feature integration (CMFA) and (ii) directionally optimized feature fusion (CAG).

## 4. Conclusions and Discussion

We proposed DSAE-Net, a lightweight dual-stage attention-enhanced network that achieves breakthrough parameter efficiency in retinal vessel segmentation through three core innovations: (1) a parametric cascaded W-shaped encoder, enabling progressive feature refinement with only 0.08 M parameters; (2) Skeleton Distance Loss (SDL), leveraging vessel topology priors to resolve severe class imbalance; and (3) coordinated attention mechanisms (CMFA + CAG) that synergistically expand receptive fields while optimizing multi-scale feature fusion.

Extensive validation on the DRIVE, CHASE_DB1, HRF, and STARE datasets demonstrates DSAE-Net’s superior trade-off between accuracy and complexity: it attains state-of-the-art or competitive segmentation metrics (Acc/AUC/Dice/MCC) while utilizing merely 1% of U-Net’s parameters and 2.2% of ConvUNeXt’s. Ablation studies confirm the critical synergies within our cascaded framework, where CMFA and CAG collectively enhance capillary recovery through global dependency modeling and directional feature reweighting.

Despite these advances, two limitations warrant further investigation: (1) occasional oversegmentation of complex vascular branches, attributed to SDL’s spatial weighting strategy, and (2) underdetection of subtle vessels in high-resolution HRF images, exacerbated by training–test resolution discrepancies. The clinical implications of these limitations are noteworthy. Oversegmentation could introduce false positives into automated screening systems, potentially leading to unnecessary referrals. More critically, the underdetection of micro-vessels, as observed in HRF, poses a greater risk, as it may result in missing early signs of diabetic retinopathy (e.g., microaneurysms) or impaired capillary perfusion indicative of glaucoma. Future work will focus on adaptive kernel selection and SDL’s spatial prior refinement—particularly optimizing its terminal vessel weighting—to enhance micro-vessel delineation without compromising efficiency. We will also explore comparisons with emerging lightweight vision transformers to further benchmark our architectural approach. These refinements are expected to solidify DSAE-Net’s applicability in clinical screening environments with diverse imaging protocols.

## Figures and Tables

**Table 1:** Summary of retinal vessel segmentation datasets used in the experiments.

Dataset	Train	Val	Test	Image Size (Pixels)	Characteristics / Purpose
DRIVE [37]	15	5	20	565×584	General benchmark; includes pathologies
CHASE-DB1 [38]	16	6	6	999×960	Images from children; demographic robustness
HRF [39]	20	5	20	3504×2336	High-resolution; healthy/DR/glaucoma subjects
STARE [40]	12	4	4	605×700	Diverse pathologies; robustness testing

**Table 2:** Model efficiency comparison: parameters, FLOPs, and inference time on different hardware.

Model	Year	Parameters (M)	FLOPs (G)	Time (CPU) (s) *	Time (GPU) (s) *
UNet	2015	7.76	54.85	0.97	0.86
Attention UNet	2018	34.88	266.53	3.55	2.59
ResUNet++	2019	4.06	63.26	1.65	1.07
SA-UNet	2020	0.48	10.61	1.02	0.71
ConvUNeXt	2022	3.51	29.01	1.53	1.02
FR-UNet	2022	5.72	235.92	5.10	3.58
PDF-UNet	2023	10.52	44.74	1.13	0.77
ULite	2023	0.88	**3.03**	**0.86**	**0.55**
MSDANet	2024	55.55	650.91	6.02	2.35
LMFR-Net	2025	**0.07**	13.97	1.05	0.70
DSAE-Net	2025	0.08	4.76	1.00	0.67

* CPU: Intel(R)Core(TM)i7-9750H CPU @ 2.60 GHZ; GPU: NVIDlA GeForce GTX 1660 Ti. **bold**: best; underline: second-best.

**Table 3:** Segmentation performance (Acc, AUC, Dice, MCC, HD95) across datasets.

Dataset	Model	Acc (%)	AUC (%)	Dice (%)	MCC (%)	HD95
DRIVE	UNet *	94.87	96.99	80.00	77.06	37.96
	Attention UNet *	95.42	**98.04**	82.32	79.74	20.03
	ResUNet++	94.94	97.12	80.18	77.28	37.75
	SA-UNet *	95.44	97.95	82.42	79.82	20.84
	ConvUNeXt *	95.46	97.76	82.31	79.71	15.63
	FR-Unet	95.36	97.72	81.72	79.06	26.54
	PDF-UNet	95.35	97.80	81.94	79.27	24.5
	ULite *	95.40	97.48	81.77	79.14	21.83
	MSDANet	95.48	97.85	82.43	79.84	27.43
	LMFR-Net	95.29	97.64	81.69	78.99	26.58
	DSAE-Net *	**95.48**	98.00	**82.44**	**79.86**	**12.12**
CHASE-DB	UNet	95.89	97.99	79.11	76.88	64.42
	Attention UNet	96.22	98.45	81.22	79.27	34.74
	ResUNet++	96.25	98.48	81.29	79.33	26.25
	SA-UNet	96.14	98.52	80.99	78.02	35.59
	ConvUNeXt	96.15	98.41	80.90	78.91	36.42
	FR-Unet	96.15	98.51	80.95	78.97	31.59
	PDF-UNet	96.07	98.36	80.63	78.62	41.17
	ULite	95.95	98.11	79.91	77.80	43.92
	MSDANet	96.14	98.09	80.89	78.90	40.75
	LMFR-Net	96.04	98.42	80.56	78.99	36.84
	**DSAE-Net**	**96.25**	**98.56**	**81.46**	**79.54**	**22.65**
HRF	UNet	96.11	97.44	78.79	76.65	170.6
	Attention UNet	**96.37**	**98.01**	**80.46**	**78.46**	127.14
	ResUNet++	95.91	96.97	77.41	75.20	225.36
	SA-UNet	96.29	97.94	80.10	78.06	128.25
	FR-Unet	96.26	97.73	79.72	77.66	140.58
	ConvUNeXt	96.21	97.57	79.41	77.33	132.63
	PDF-UNet	96.27	97.76	79.81	77.75	144.42
	ULite	96.04	97.24	78.35	76.19	151.5
	MSDANet	96.26	97.70	79.92	77.86	150.3
	LMFR-Net	96.30	97.84	80.03	77.98	147.2
	**DSAE-Net**	96.31	97.90	80.12	78.10	126.25
STARE	UNet	96.45	97.87	76.60	75.62	57.25
	Attention UNet	96.64	97.85	79.02	77.53	49.75
	ResUNet++	96.61	98.15	80.01	78.19	38.38
	SA-UNet	96.70	98.55	80.71	78.92	30.67
	ConvUNeXt	**96.80**	98.34	81.53	79.78	27.00
	FR-Unet	96.73	98.48	79.56	78.13	35.75
	PDF-UNet	96.44	98.32	80.05	78.13	30.27
	ULite	96.54	97.86	80.01	78.12	**24.5**
	MSDANet	96.19	98.07	79.33	77.40	30.33
	LMFR-Net	96.52	97.91	79.75	77.85	35.25
	**DSAE-Net**	96.68	**98.73**	**81.92**	**80.25**	27.25

* Results marked with * are obtained via 5-fold cross-validation. Mean values and standard deviations for UNet, Attention UNet, SA-UNet, ConvUNeXt, ULite, and DSAE-Net based on 5-fold cross-validation are provided in Appendix A, Table A1. **bold**: best; underline: second-best.

**Table 4:** Average performance of DSAE-Net for each of the datasets in Table 1 over 5 training runs, with a confidence interval containing the true mean with probability *p* = 95%, under a normality assumption for performance.

Dataset	Acc (%)	AUC (%)	Dice (%)	MCC (%)
DRIVE	95.48 ± 0.02	98.00 ± 0.04	82.44 ± 0.11	79.86 ± 0.12
CHASE-DB	96.25 ± 0.03	98.56 ± 0.02	81.46 ± 0.22	79.54 ± 0.23
HRF	96.31 ± 0.04	97.90 ± 0.05	80.12 ± 0.08	78.10 ± 0.09
STARE	96.68 ± 0.03	98.73 ± 0.02	81.92 ± 0.13	80.25 ± 0.15

**Table 5:** Hyperparameter ablation: T: (*k*, *m*) scaling on DRIVE.

Metric	Configuration: Depth *k*/Width *m*					
	3/4	3/8	3/16	4/8	4/16	4/32
Param (M)	0.02	0.08	0.31	0.32	1.27	5.03
FLOPs (G)	1.33	4.76	18.57	6.81	26.23	102.93
Dice (Val) (%)	81.94	82.82	83.08	82.95	83.40	83.10
AUC (Val) (%)	98.03	98.18	98.26	98.19	98.36	98.30
Dice (Test) (%)	81.77	82.44	82.31	82.41	82.68	81.98
AUC (Test) (%)	97.71	98.00	97.76	98.01	98.04	97.74

Note: Dice/AUC reported in percentage points for cross-metric consistency.

**Table 6:** Sensitivity analysis of the balancing factor α in the combined loss function on the DRIVE dataset.

Metric	Value of α			
	0.0	0.3	0.7	1.0
Dice (Val) (%)	82.77	82.81	82.82	82.8
HD95 (Val)	21.0	17.7	10.5	9.0
Dice (Test) (%)	82.40	82.41	82.44	82.44
HD95 (Test)	23.05	20.45	12.68	15.35

**Table 7:** Ablation study: module contributions on DRIVE and CHASEDB1 datasets.

Dataset	Model	Param (M)	Acc	AUC	Dice	MCC
DRIVE	w/o CAG & CMFA	0.06	94.99	97.11	81.20	78.26
	w/o CAG	0.06	95.17	97.49	81.22	78.46
	w/o CMFA	0.08	95.46	97.95	82.20	79.59
	Full	0.08	95.48	98.00	82.44	79.86
CHASE-DB	w/o CAG & CMFA	0.06	96.02	98.12	79.24	77.01
	w/o CAG	0.06	96.22	98.51	81.16	79.18
	w/o CMFA	0.08	96.24	98.69	81.44	79.52
	Full	0.08	96.25	98.56	81.46	79.54

## Data Availability

The Drive and CHASE-DB1 datasets, along with the HRF dataset, are publicly available at https://gitcode.com/open-source-toolkit/33a91 (accessed on 1 September 2025). The STARE dataset is available at http://cecas.clemson.edu/~ahoover/stare/ (accessed on 1 September 2025). The code for DSAE-Net is available at https://anonymous.4open.science/r/DSAE-Net-2CB1 (accessed on 1 September 2025).

## Appendix A

**Table A1:** The mean and standard deviation results from 5-fold cross-validation for UNet, Attention UNet, SA-UNet, SA-UNet, ConvUNeXt, ULite, and DSAE-Net.

Model	Acc (%)	AUC (%)	Dice (%)	MCC (%)	HD95
UNet	94.87 ± 0.08	96.99 ± 0.12	80.00 ± 0.43	77.06 ± 0.45	37.96 ± 2.96
Attention UNet	95.42 ± 0.13	98.04 ± 0.07	82.32 ± 0.09	79.74 ± 0.11	20.03 ± 1.98
SA-UNet	95.44 ± 0.09	97.95 ± 0.12	82.42 ± 0.08	79.82 ± 0.12	20.84 ± 1.24
ConvUNeXt	95.46 ± 0.12	97.76 ± 0.10	82.31 ± 0.20	79.71 ± 0.26	15.63 ± 2.66
ULite	95.40 ± 0.27	97.48 ± 0.29	81.77 ± 0.44	79.14 ± 0.61	21.83 ± 2.92
DSAE-Net	95.48 ± 0.02	98.00 ± 0.04	82.44 ± 0.10	79.86 ± 0.11	12.12 ± 2.17
